# Physiologically-Based Pharmacokinetic Modelling to Predict the Pharmacokinetics and Pharmacodynamics of Linezolid in Adults and Children with Tuberculous Meningitis

**DOI:** 10.3390/antibiotics12040702

**Published:** 2023-04-03

**Authors:** Carlijn H. C. Litjens, Laurens F. M. Verscheijden, Elin M. Svensson, Petra H. H. van den Broek, Hedwig van Hove, Jan B. Koenderink, Frans G. M. Russel, Rob E. Aarnoutse, Lindsey H. M. te Brake

**Affiliations:** 1 Department of Pharmacy, Radboud Institute for Health Sciences, Radboud University Medical Center, Geert Grooteplein Zuid 10, 6525 GA Nijmegen, The Netherlands; 2 Department of Pharmacology and Toxicology, Radboud Institute for Molecular Life Sciences, Radboud University Medical Center, Geert Grooteplein Zuid 10, 6525 GA Nijmegen, The Netherlands; 3 Department of Pharmacy, Uppsala University, 75123 Uppsala, Sweden

**Keywords:** PBPK, linezolid, tuberculous meningitis

## Abstract

Linezolid is used off-label for treatment of central nervous system infections. However, its pharmacokinetics and target attainment in cranial cerebrospinal fluid (CSF) in tuberculous meningitis patients is unknown. This study aimed to predict linezolid cranial CSF concentrations and assess attainment of pharmacodynamic (PD) thresholds (AUC:MIC of >119) in plasma and cranial CSF of adults and children with tuberculous meningitis. A physiologically based pharmacokinetic (PBPK) model was developed to predict linezolid cranial CSF profiles based on reported plasma concentrations. Simulated steady-state PK curves in plasma and cranial CSF after linezolid doses of 300 mg BID, 600 mg BID, and 1200 mg QD in adults resulted in geometric mean AUC:MIC ratios in plasma of 118, 281, and 262 and mean cranial CSF AUC:MIC ratios of 74, 181, and 166, respectively. In children using ~10 mg/kg BID linezolid, AUC:MIC values at steady-state in plasma and cranial CSF were 202 and 135, respectively. Our model predicts that 1200 mg per day in adults, either 600 mg BID or 1200 mg QD, results in reasonable (87%) target attainment in cranial CSF. Target attainment in our simulated paediatric population was moderate (56% in cranial CSF). Our PBPK model can support linezolid dose optimization efforts by simulating target attainment close to the site of TBM disease.

## 1. Introduction

Linezolid is an antibiotic in the oxazolidinone class and was approved in 2000 by the US Food and Drug Administration (FDA) [1]. The World Health Organization (WHO) recently re-classified linezolid as a Group A drug for treatment of multidrug-resistant tuberculosis (MDR-TB) [2]. Although there is evidence from randomized controlled trials for efficacy of linezolid in drug-resistant pulmonary tuberculosis (TB) [3], there is a lack of comparative data on efficacy and safety for its use in extrapulmonary types of TB, such as tuberculous meningitis (TBM).

TBM only makes-up 1–2% of all TB cases, but it is the most severe manifestation of tuberculosis, resulting in death or disabilities in up to 50% of the affected patients. Furthermore, TBM is particularly prevalent in young children under the age of five years [4]. Linezolid has been used in the management of critically ill adults with bacterial central nervous system (CNS) infections, resulting in mean cerebrospinal fluid (CSF)/plasma ratios between 0.56 and 0.8 [5,6,7,8]. Unfortunately, linezolid plasma and CNS pharmacokinetic (PK) and pharmacodynamic (PD) data are limited for TBM. A recently published phase 2A trial (LASER-TBM) showed no significant difference in the incidence of specific adverse events or death between linezolid containing treatment arms versus standard of care, but no PK data were reported [9]. One retrospective analysis studied the clinical impact of (short-term) supplementation of linezolid in a dose of 600 mg twice daily (BID) to a background TBM regimen, and results suggested a therapeutic benefit for life-threatening TBM, but again no PK data were reported [10]. Recently, one publication reported linezolid concentrations in plasma and CSF, but only for two time points (2 and 6 h post dose), showing a promising CNS penetration rate of ~50% in TBM patients [11].

In clinical trials and practice, doses of linezolid varying from 300 and 600 mg BID to 1200 mg once daily (QD) orally are being used to treat TB [3,9,11], continuously balancing between optimal efficacy and adverse effects, such as peripheral neuropathy and bone marrow suppression [12,13]. PK–PD analyses based on plasma or serum measurements allow for evidence-based dose and exposure optimization of antibiotics. Ideally, exposures at the site of infection are also considered, but pharmacokinetic profiles in TBM infection sites, including the brain, meninges, and intracranial CSF, are difficult to obtain. PK–PD analyses in TBM so far have mostly focused on intensive PK sampling in the plasma compartment, as well as single time point spinal CSF concentrations in the CNS compartment [11,14,15]. While such single time point concentrations provide an indication of the degree of CNS exposure, their representativeness for total exposure and target attainment at the site(s) of infection can be debated [16]. Physiologically based pharmacokinetic (PBPK) modelling is a mechanistic approach in which system-specific parameters and drug-specific parameters are combined to predict absorption, distribution, metabolism, and elimination, as well as the overall PK profile of a drug in different compartments, including brain tissue and cranial and spinal CSF [17,18]. In addition, a PBPK model developed for adults can be extrapolated to other populations at risk, such as children, by taking into account the effect of age-related differences in brain physiological parameters on predicted PK profiles [19].

In this study, a CNS PBPK model was developed to predict the pharmacokinetics of linezolid in the CNS of adults and children with TBM and to assess whether currently investigated linezolid doses achieve sufficient cranial CSF drug exposures.

## 2. Materials and Methods

### 2.1. Development of a Linezolid PBPK Model

**Figure 1:**
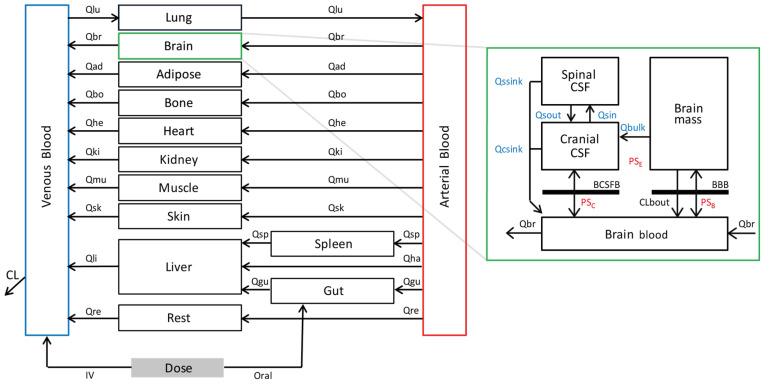
Schematic outline of the PBPK model, including four CNS compartments, adapted from Verscheijden et al. (2019) [20], with permission. Qsin and Qsout represent CSF shuttle flow between cranial CSF and spinal CSF compartments. Qssink and Qcsink are the flows from CSF compartments to blood. Qbulk represents bulk flow from brain mass to cranial CSF. PSB, PSC, and PSE represent permeability surface area products between brain blood and brain mass, brain blood and cranial CSF, and brain mass and cranial CSF, respectively. CLbout represents overall clearance of efflux transporters P-gp and BCRP expressing at BBB. Subscripts lu, br, ad, bo, he, ki, mu, sk, li, re, gu, sp, and ha denote lung, brain, adipose tissue, bone, heart, kidney, muscle, skin, liver, rest tissue, gut, spleen, and hepatic artery, respectively. CL is the total clearance from the model. IV is an intravenous dose, and oral is an oral dose route of administration.

A linezolid 4-compartment permeability-limited CNS PBPK model was developed using Simcyp simulator software Version 19 Release 1 (Certara UK Limited, Simcyp Division, Sheffield, UK), based on a previously published brain model [20] which was able to simulate plasma and CSF pharmacokinetics well. The model was refined using available data in the literature and experiments on transport of linezolid across the blood–brain barrier. A schematic overview and description of the CNS PBPK model can be found in Figure 1. A full description can be found in Supplementary File S1, and an overview of parameter values used in the final model can be found in Supplementary Table S1.

### 2.2. Transporter-Mediated Clearance of Linezolid

In order to build the PBPK model, molecular pharmacological studies were performed in-house to identify the transporters involved in linezolid efflux at the blood–brain barrier. Human embryonic kidney 293 (HEK293) cells overexpressing a single efflux transporter present at the blood–brain barrier (P-gp, BCRP, MRP1, 4 and 5) [21] were used to determine the involvement of these transporters in the brain disposition of linezolid. Experiments were performed identically, as described previously by Te Brake et al. (2016) [22] at a concentration of 100 µM linezolid. Linezolid was confirmed as a substrate for BCRP, but not for P-gp, MRP1, MRP4, and MRP5 (Supplementary Figure S1). Due to the clinical drug–drug interaction between linezolid and rifampicin, resulting in about 30% lower linezolid concentrations [23], and the inductive effect of rifampicin on P-gp [24], it has been suggested previously that linezolid is a substrate for P-gp [25]. Although others, including the manufacturer, report that linezolid is not a substrate for P-gp [1]. Therefore, transporter-mediated clearance by both MDCKII-BCRP and MDCKII-P-gp cells was tested. A detailed description of the experiments can be found in Supplementary File S2.

### 2.3. Evaluation of the CNS Model-Plasma and CSF Exposures

The performance of the CNS model was evaluated based on human linezolid plasma and cranial CSF exposure data after single and multiple intravenous doses (600 mg in adults and 10 mg/kg in children) that were extracted from literature. An overview of these studies can be found in Supplementary Table S2. All the literature studies used in this evaluation are related to patients with other CNS infections, as opposed to those caused by TB. For cranial CSF simulations in critically ill patients, the performance of the model was checked by visual inspection of the PK curves, as well as comparison of the simulated PK parameter AUC_0–12_ with the reported median values in each study. Acceptance criteria for the model were defined as an AUC_0–12_ not deviating more than two-fold (0.5–2.0×) from the median observed AUC_0–12_ values reported in the literature [26]. The percent penetration of linezolid into cranial CSF was calculated by mean predicted AUC_0–12-cranial CSF_/mean predicted AUC_0–12-serum._

### 2.4. Extrapolation of the CNS Model-Target Attainment in TB Patients

The developed CNS model was used to predict cranial CSF exposure based on plasma curves of actual pulmonary TB patients, as reported in the literature. An overview of these studies can be found in Supplementary Table S2. To estimate target attainment, the efficacy predicting parameters, so called pharmacokinetic-pharmacokinetic (PK–PD) indices, AUC_0–24_ over the minimum inhibitory concentrations (AUC_0–24_:MIC ratio, with a reported target range of 100–119 [12,27], and percentage time above MIC (T > MIC should be >80%) [27], were evaluated for various multiple dose simulations, i.e., 300 mg oral linezolid BID, 600 mg oral BID, and 1200 oral QD in adults and ~10 mg/kg oral BID in children [28,29,30,31,32]. As the simulated AUC_0–12_ for twice daily dosing (BID) was determined at steady state (≥4 doses), we assumed that the AUC_0–24_ was double the AUC_0–12_ [32]. The reported MIC in adult TB studies ranged between 0.125 and 1 mg/L, and the latter was used as a “worst-case” threshold value [30,31,32].

## 3. Results

### 3.1. Transporter-Mediated Clearance of Linezolid

Linezolid was confirmed to be a substrate for BCRP, but not for P-gp, MRP1, MRP4, or MRP5 (Supplementary Figure S1). Due to the drug–drug interaction between linezolid and rifampicin, as well as the inductive effect of rifampicin on P-gp [24], it has been suggested previously that linezolid is a substrate for P-gp [25]. Therefore, transporter-mediated clearance by both MDCKII-BCRP and MDCKII-P-gp cells was tested. The net efflux ratios for BCRP and P-gp were 3.59 and 1.43, and the CL_efflux,vitro_ values were 16 and 2.1 µL/min/mg protein, respectively. The inclusion of BCRP and P-gp in the BBB of the CNS model had only a modest effect on the calculated AUC (AUC_no transporters_/AUC_transporters_ = 1.1).

### 3.2. Evaluation of the CNS Model–Plasma and CSF Exposures

**Figure 2:**
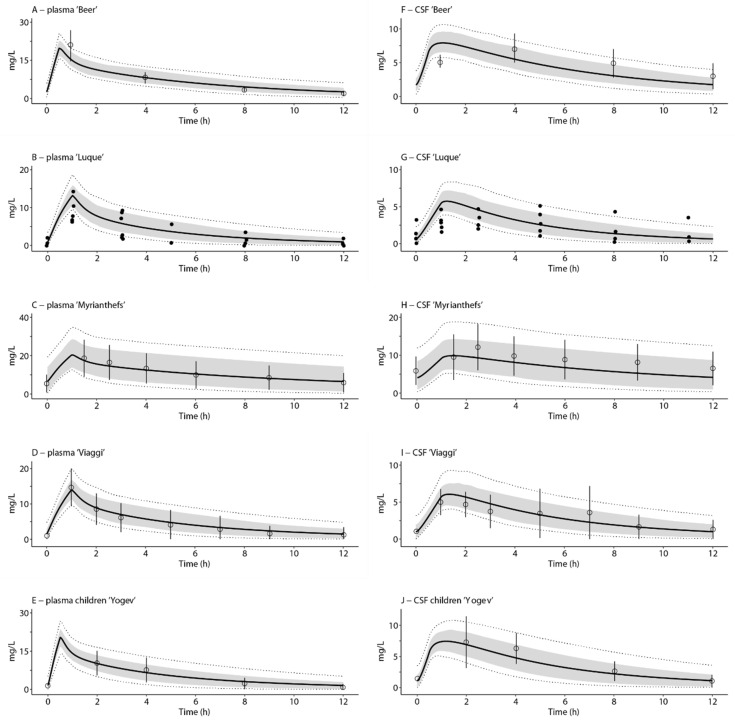
Simulations of linezolid concentration–time profiles in plasma (**A**–**E**) and cranial CSF (**F**–**J**) in critically ill adult and paediatric patients. Figures relate to intravenous administration of 600 mg linezolid twice daily for >two days in adults, as well as 10 mg/kg intravenous administration twice daily for two days in children (0.25–21 years). The *x*-axis indicates time after the last dose. The solid black lines indicate mean simulated profiles, the grey areas represent the area between the 16th and 84th percentile (equal to the standard deviation) of the mean, and the dotted lines indicate the 5th and 95th percentiles of the mean. Closed dots indicate measured individual data derived from the literature [6], and open circles indicate the mean with reported standard deviation derived from the literature [5,7,8,33]. The names in the headings of the figures relate to the first authors of the papers from which clinical data points were derived.

Optimized plasma PK curves, i.e., by using the literature-based plasma clearance values, as well as predicted cranial CSF and brain concentration-time curves after single dose linezolid, can be found in Supplementary Figure S2. Optimized linezolid plasma PK curves and predicted cranial CSF curves after multiple doses in critically ill adults (600 mg i.v. BID for >two days) and children (~10 mg/kg i.v. BID for two days) are displayed in Figure 2**.** All steady-state cranial CSF AUC-ratios were within the two-fold range (0.71–1.37) of the ratios reported in the literature [5,6,7,8,33]. The mean predicted cranial penetration ratio at steady-state was 0.69. A predicted spinal CSF curve can be found in Supplementary Figure S3.

### 3.3. Extrapolation of the CNS Model-Target Attainment in TB Patients

**Figure 3:**
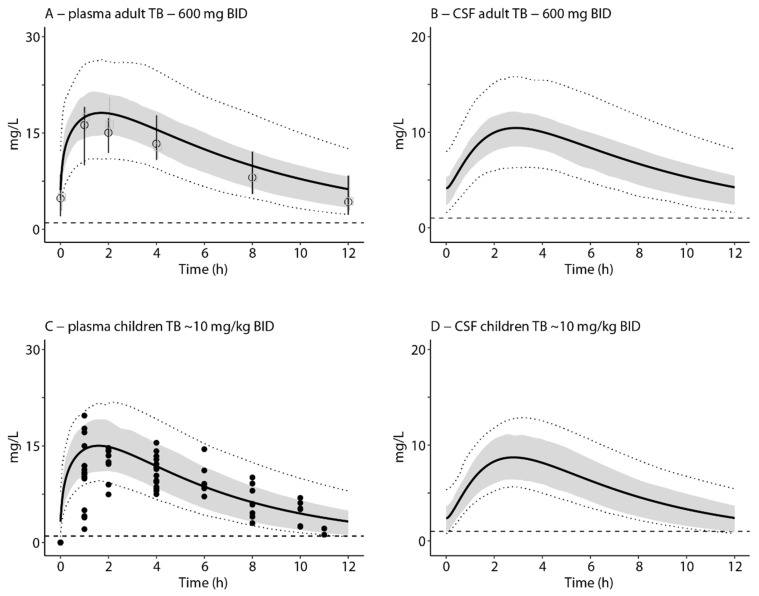
Simulations of linezolid concentration–time profiles in plasma and cranial CSF in adults (**A**,**B**) and paediatric (**C**,**D**) TB patients. The results relate to oral administration of 600 mg (adults) or 9.24 mg/kg (children; 0.6–9.4 years) linezolid twice daily. The *x*-axis indicates time after the last dose. The solid black lines indicate simulation of the mean profile, the grey areas represent the area between the 25th and 75th percentile (**A**,**B**) or 16th and 84th percentile (equal to the standard deviation) of the mean (**C**,**D**), and the dotted lines indicate the 5th and 95th percentiles of the mean. Open black circles and open light grey squares indicate median with interquartile range from the literature [30,31]. Closed dots indicate measured individual data derived from literature [29]. Dashed horizontal line indicate the MIC value of 1 mg/L linezolid for *Mycobacterium tuberculosis* [30,31]. In the clinical studies, linezolid was provided in addition to standard care.

Since all predictions of cranial CSF concentrations in critically ill patients were within the accepted ranges, optimized plasma PK curves of pulmonary TB patients [29,30,31] were used to predict and evaluate the PK–PD indices of interest for *Mycobacterium tuberculosis* (Figure 3). Simulation of multiple oral doses of 600 mg BID in adults resulted in a geometric mean predicted total plasma AUC_0–24_:MIC ratio of 281 and a mean predicted cranial AUC_0–24_:MIC ratio of 181. A total of 99% and 87% of the simulated population reached the AUC:MIC threshold of 119 in plasma and cranial CSF, respectively. Simulation of ~10 mg/kg BID in children resulted in a geometric mean predicted total plasma AUC_0–24_:MIC ratio of 202, and 93% of the simulated population reached the PD threshold. The mean predicted cranial CSF AUC_0–24_:MIC ratio was 135 and only 56% of the simulated paediatric population that reached the threshold for cranial CSF. Mean total plasma and cranial CSF concentration-time curves were all above the worst case MIC of 1 mg/L for the complete dosing interval (Table 1).

**Figure 4:**
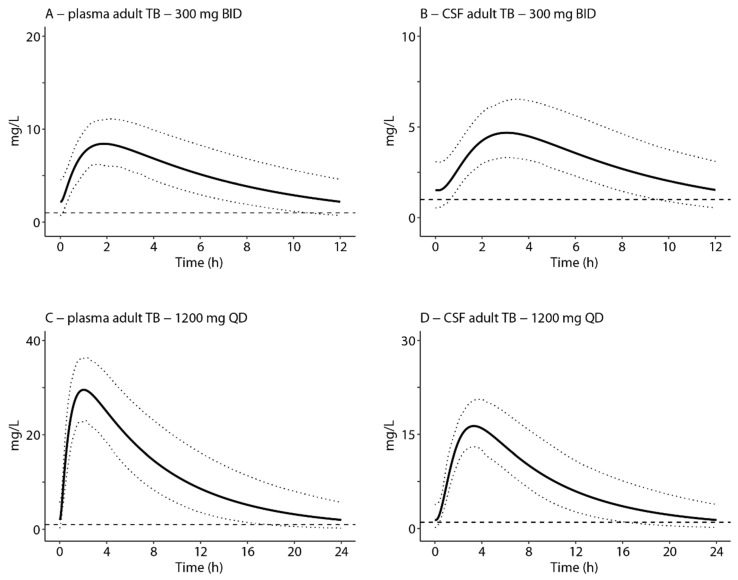
Simulations of linezolid concentration–time profiles in plasma and cranial CSF after 300 mg BID (**A**,**B**) and 1200 mg QD (**C**,**D**) in adult TB patients. The *x*-axis indicates time after the last dose. The solid black lines indicate simulation of the mean profile, and the dotted lines indicate the 5th and 95th percentiles of the mean. Simulations were performed based on plasma PK parameters values in adult TB patients as described by Diacon et al. (2020) [32]. Dashed horizontal lines indicate the MIC value of 1 mg/L linezolid for *Mycobacterium tuberculosis* [30,31]. In the clinical study provided, linezolid was provided as a monotherapy.

Simulation of plasma and cranial CSF after five doses of 300 mg BID and 1200 mg QD, assuming plasma clearance as described by Diacon et al. [32], in adults, resulted in a geometric mean plasma AUC_0–24_:MIC of 118 and 262 for the 300 mg BID and 1200 mg QD regimen, respectively. In the respective dosing regimens, 45% and 99% of the simulated population reached the threshold AUC_0–24_:MIC ratio of 119. The mean cranial AUC_0–24_:MIC for the 300 mg BID regimen was 74, and only 4% of the simulated population reached the threshold AUC_0–24_:MIC ratio. For the 1200 mg QD regimen, the mean cranial AUC_0–24_:MIC ratio was 166, and 87% reached the threshold ratio of 119. Mean concentration–time curves were all above the MIC of 1 mg/L for the complete dosing interval (Figure 4 and Table 1).

Table 1 gives an overview of predicted PK–PD indices for the various simulated dosing regimens (in adult and paediatric TBM populations).

## 4. Discussion

We developed a CNS PBPK model based on the available PK data in the literature and in-house experiments on transport of linezolid across the blood–brain barrier. We first confirmed the performance of the CNS model in critically ill patients (non-TB CNS infections), and we subsequently used the model to predict cranial CSF linezolid concentrations in adults and paediatric TB patients with population specific plasma clearance values. Model-based predictions were able to demonstrate that mean cranial CSF exposure values reach *Mycobacterium tuberculosis* PK–PD thresholds, i.e., an AUC_0–24_:MIC ratio of >119 and >80% of the dosing interval above the MIC (MIC set at 1 mg/L), when dosed at 600 mg BID and 1200 mg QD in adults, as well as ~10 mg/kg BID in children. However, in our simulations, 13% of the adult (600 mg BID and 1200 mg QD), and 44% of the paediatric simulated population (~10 mg/kg), still did not reach the PK–PD threshold in cranial CSF (Table 1).

The optimal dosage of linezolid in MDR-TB is still under debate, mainly because of toxicity related concerns, such as myelosuppression and peripheral neuropathy, after prolonged use of linezolid (>28 days). Currently, a total daily dose of 600 mg seems to offer a good balance between efficacy and toxicity in MDR-TB [12,13], but the optimal dosage may be different in TBM. First, longer term toxicity concerns are less relevant for linezolid use in TBM, since addition of linezolid to the standard TBM regimen is intended only for the first two to four weeks of treatment when the mortality rate is at its highest [34,35,36]. Second, although efficacy in plasma in MDR-TB patients seems to be adequate after a total daily dose of 600 mg, this dose might be inadequate for TBM patients, since only about ~50% of linezolid seems to cross the blood–brain barrier. Lastly, in contrast to MDR-TB, patients with (drug-susceptible) TBM are treated with rifampicin or high dose rifampicin [15], which induces linezolid clearance and results in ~30% reduced linezolid plasma concentrations [23]. Therefore, in this study, a broad range of oral linezolid dosing regimens (i.e., 300 mg BID, 600 mg BID and 1200 mg QD) in plasma and cranial CSF in adults were evaluated. For these simulations, we used a worst-case MIC of 1 mg/L (reported range 0.125–1 mg/L) [30,31,32,37] and a target for the most important PD parameter AUC_0–24_:MIC of >100–119 [12,27].

In order to build our PBPK model, we performed additional molecular pharmacological experiments to assess transporter-mediated clearance values for linezolid at the blood–brain barrier, as these were missing in the literature. By using HEK293 cells and MDCKII cells overexpressing a single efflux transporter, we showed that linezolid is a weak substrate for BCRP, and it is an even weaker substrate for P-gp. The effect of these transporter values on blood–brain barrier permeability in the PBPK model was limited, suggesting that passage of the BBB is mostly dependent on passive diffusion.

Our model-based approach proved to be informative, but it still suffered from limitations. First, clearance could not be mechanistically incorporated in the model. This is because the clearance pathways of linezolid are not fully unravelled yet and appear largely independent from classic phase I/II metabolism. Plasma clearance values from the literature were used per study simulation to optimally reflect clinically observed values. In the studies reporting PK values of critically ill patients used for verification of the CNS model, clearance values ranged between 7.3 and 16.6 L/h [5,6,7,8]. However, values up to >30 L/h have been reported in patients with augmented renal clearance [38]. As a result, it is difficult to predict linezolid clearance a priori without use of measured plasma concentrations, especially when linezolid is combined with rifampicin. Since clearance is reported to be concentration dependent, it should also be re-evaluated after a dose change [39]. Second, we modelled a Caucasian healthy volunteer population, rather than critically ill patients or Asian/African/South-American TBM populations, as these populations are not validated in Simcyp. Nevertheless, differences in volume of distribution and plasma clearance were accounted for. Third, for the evaluation of target attainment, we assumed the plasma clearance in TBM patients to be comparable to pulmonary TB patients, as plasma linezolid clearance values in TBM patients are currently missing. Lastly, it might be debated whether plasma PK–PD indices can be directly translated to CSF. The plasma AUC is based on total (protein bound plus unbound) concentrations, while drugs in the CSF are most likely largely unbound, since the protein content of CSF is much lower than that of plasma [40]. However, plasma protein binding of linezolid is only ~30%, reducing the potential impact of protein binding on the PK–PD index target. Of note, inflamed meninges are assumed to be more permeable, even though Yogev et al. (2010) suggested that the penetration of linezolid in critically ill children with and without inflamed meninges did not differ [33].

Our CNS PBPK model can be used to predict cranial CSF disposition of linezolid in case plasma concentrations and corresponding clearance values are available. The validity of these predictions could then be confirmed by comparing our modelled CSF disposition results with actual spinal/cranial CSF linezolid exposures in TBM patients. Unfortunately, combined plasma and total CSF exposure data are not yet available for linezolid. Recently, linezolid plasma and CSF concentrations were reported in 17 TBM patients, but only for two time points (2 and 6 h post dose), so total exposure or plasma clearance could not be calculated [11]. At the moment, multiple trials with PK substudies are ongoing, which investigate a 1200 mg daily dose in various regimen combinations: INTENSE TBM [41], the ALTER study, the SIMPLE study, and IMAGINE TBM. PK–PD analyses from these studies are essential to allow for evidence-based dose selection and exposure target optimization of linezolid. Plasma and CSF PK data of these studies can be used to complement and validate our PK profiles and target attainment estimates at difficult-to-sample sites of TBM disease, including brain and intracranial CSF.

## 5. Conclusions

Our CNS PBPK model predicted cranial CSF disposition and target attainment in TBM patients, using plasma clearance values observed in pulmonary TB patients. Our simulations suggest acceptable CNS exposure following a total daily dose of 1200 mg, with PK–PD thresholds in cranial CSF being reached in 87% of the simulated adult population. In our paediatric population (~10 mg/kg BID), simulated target attainment in cranial CSF was only moderate (56%). Our model can be used to predict cranial CSF disposition in case plasma concentration measurements of a patient population are available. These simulations can complement ongoing linezolid dose optimization efforts by providing target attainment estimates at difficult-to-sample sites of TBM disease.

## Figures and Tables

**Table 1:** Predicted PK–PD indices per dosing regimen.

Dose	AUC_0–24_/MIC Plasma ^a^	Reaching Threshold Plasma (%) ^b^	AUC_0–24_/MIC Cranial CSF ^c^	Reaching Threshold Cranial CSF (%) ^b^	T > MIC (%) ^d^
**Adults**					
300 mg BID oral	118 (75–189)	45	74 (45–117)	4	100
600 mg BID oral	281 (168–491)	99	181 (99–300)	87	100
1200 mg QD oral	262 (166–423)	99	166 (100–266)	87	100
**Children**					
~10 mg/kg BID oral	202 (117–360)	93	135 (74–225)	56	100

^a^ geometric mean (5th–95th percentile) AUC_0–24 h_ at steady state, ^b^ threshold AUC/MIC of 119, ^c^ mean (5th–95th percentile) AUC_0–24 h_ at steady state, ^d^ T > MIC at steady state for plasma and cranial CSF. AUC: area under the concentration-time curve, MIC: minimum inhibitory concentration, CSF: cerebrospinal fluid.

## Data Availability

The data that support the findings of this study are available from the corresponding author upon reasonable request.

## Supplementary Materials

The following supporting information can be downloaded at: https://www.mdpi.com/article/10.3390/antibiotics12040702/s1. File S1: Linezolid PBPK model. File S2: General cell culture and integrity of MDCK-II monolayers. Table S1: Summary of physicochemical, in vitro, and in vivo linezolid parameters. Table S2: Overview of clinical studies used for the validation of the simulated PK curves. Figure S1: Transport of linezolid in transiently transduced HEK293 cells. Figure S2: Single dose simulations of 600 mg intravenous linezolid concentration-time profiles in plasma, cranial CSF and brain tissue in critically ill adult patients and plasma and cranial CSF after a single dose linezolid of 10 mg/kg in critically ill children. Figure S3: Single dose simulations of 600 mg intravenous linezolid concentration-time profiles in spinal CSF in critically ill adult patients. References [1,5,6,7,8,18,23,29,30,31,32,33,39,42,43,44,45,46,47,48,49,50,51,52] are cited in Supplementary Materials.
