# Supplementary material for: Physiologically-Based Pharmacokinetic Modelling to Predict the Pharmacokinetics and Pharmacodynamics of Linezolid in Adults and Children with Tuberculous Meningitis
Source: Antibiotics (Basel). 2023 Apr 3;12(4):702. doi: 10.3390/antibiotics12040702 (PMC10135070; doi:10.3390/antibiotics12040702)
Supplement: Supplementary file 1 [file antibiotics-12-00702-s001.zip › antibiotics-2298930-supplementary.pdf]

Supplementary file S1

Linezolid PBPK model

Simulations for adults were performed using Simcyp's virtual population of healthy North European Caucasian volunteers. The pediatric population of Simcyp was used for simulations in children, which incorporates age-related differences in brain volume, brain blood flow, CSF production rate, CSF volumes and blood-brain barrier and blood-cerebrospinal fluid barrier surface area. Each simulation was performed in 100 virtual individuals. Literature reported clearance values were used to match linezolid plasma concentrations observed in studies used for validation. The age range, proportion females and dose of the simulation were matched with the clinical data set used for validation. For calculation of all the predicted area under the concentration-time curves (AUC) a linear up log down calculation method was used.

For linezolid simulations in critically ill patients, dosing occurred via the intravenous route and absorption was not incorporated in the model. For the TB(M) patient simulations, either the absorption constant (k_a) as reported by Alffenaar *et al*. (2010) was used (0.9) [1], or was predicted by the Advanced Dissolution, Absorption and Metabolism (ADAM) model based on the passive permeability of linezolid ($9.7 x 10^{-6}$ cm/s) and propranolol ($9.9 x 10^{-6}$ cm/s; for experimental details, see **Supplementary file S2**), resulting in a k_a of 1.20 to optimally reflect the plasma concentration. For the simulations in the critically ill, volume of distribution (V_{ss}) was set at 0.66 L/kg [2], whereas for the TB(M) simulations, volume of distribution was optimized to the reported V_{ss} to account for weight loss in those patients. For adults the mean reported V_d was 28.5 L, with a mean weight of ~60 kg, resulting in a V_d of 0.47 L/kg, however, mean weight in Simcyp is ~75 kg, resulting in a corrected V_d of 0.4 L/kg [1, 3]. Linezolid is described to be renally cleared for 35%, and the remaining 65% is suggested to be metabolized by non-enzymatical chemical oxidation via reactive oxygen species (ROS) and thereby indirectly dependent on NADPH [2, 4]. Reported plasma clearance values show large interindividual variabilities [5]. Due to this large variability and an uncertain contribution of the

renal clearance component per study, we decided to incorporate total plasma clearance within the parameter ‘enzyme kinetics - additional clearance’ and use the reported value (with the corresponding % coefficient of variation (CV) if available) per study to optimally reflect measured plasma values. For children, clearance was optimized by visual inspection. Based on these optimized plasma PK curves, prediction of cranial CSF per study was performed. Passive permeability-surface area product on the BBB (PSB) was optimized based on the single dose study of Viaggi *et al.* [6] and the passive permeability-surface area product on the CSF barrier (PSC) was assumed to be half of the PSB [7]. These values were not changed for the multiple dose simulations in critically ill and TBM patients. Clinical studies describing plasma, cranial and/ or spinal CSF and brain concentration-time profiles of linezolid were used for model verification and, if necessary, extracted from the original publication using WebPlotDigitizer version 4.1. An overview of the studies can be found in **Supplementary Table S2**.

Supplementary file S2

General cell culture and integrity of MDCK-II monolayers

Stable overexpressing BCRP or P-gp Madin-Darby Canine Kidney (MDCK) II cells were kindly provided by the Netherlands Cancer Institute (NKI, Amsterdam, The Netherlands). Cells were maintained at 37°C and 5% CO_2 in Dulbecco’s Modified Eagle Medium (DMEM) with 1% GlutaMAX® (Thermo Fisher, Waltham, MA, USA) and 10% fetal calf serum (FCS). At confluence, cells were seeded at a density of 30,000 per 24-well Transwell® (0.4 µm pore polyester membrane, Corning®, Tewksbury, MA, USA), and cultured for four days. The medium was refreshed one day before the experiment. To confirm the integrity of the MDCK monolayer, the permeability of lucifer yellow (50 µM) was measured over 30 minutes. The lucifer yellow apparent permeability of MDCKII-BCRP was 1.36×10^{-6} cm/s and 0.98×10^{-6} cm/s for MDCKII-P-gp, which is in line with previous reported values [8, 9].

Transporter-mediated clearance of linezolid

To determine the active transport of linezolid by BCRP and P-gp at the BBB, MDCKII-BRCP or MDCKII-P-gp cells were incubated with Hank's Balanced Salt Solution (37°C) supplemented with 10 mM HEPES (HBSS-HEPES, pH 7.4) supplemented with linezolid (100 µM) in either the apical or basolateral compartment of Transwell® tissue culture chambers. To confirm the contribution of P-gp and BCRP (i.e. by correcting for passive permeability), the same setup was used in the presence of the inhibitor KO143 (1 µM, BCRP inhibitor) or PSC833 (10 µM, P-gp inhibitor) added in the apical chamber. The passive permeability of the reference compound propranolol was determined by adding propranolol (10 µM) to the apical chamber with inhibited MDCKII-BCRP cells. In all conditions, cells were incubated for 60 minutes in an orbital shaker (120 rounds per minute (RPM)) at 37°C. Linezolid, propranolol, KO143 and PSC833 were purchased from Merck (Darmstadt, Germany). The internal standard d_3-linezolid (Alsachim, Illkirch-Graffenstaden, France; end concentration 25 ng/ml) was added and all samples were centrifuged at 13,200 RPM for three minutes for protein precipitation. Linezolid was measured using liquid chromatography mass spectrometry (LC-MS/MS) consisting of an ultraperformance liquid chromatograph (UPLC; Acquity I-class; Waters, Milford, MA, USA) coupled to a Xevo TQ-S micro (Waters, Milford, MA, USA). Separation was performed with LC at 40°C using a BEH C18 column (1.7µm 50x2.1mm; Acquity UPLC; Waters, Ireland). The mobile phase consisted of solvent A (1mM NH_4FA + 0,1% [vol/vol] formic acid [HCOOH] in water) and solvent B (0,1% [vol/vol] formic acid [HCOOH] in acetonitrile) at a flow rate of 0.35 ml/min using the following gradient: 0 to 2 min from 100% solvent A to 50% A and 50% B and between 2 to 4 min 100% solvent A.

The apparent permeability (P_{app}) was calculated using equation 1 [10], and the efflux ratio (ER) was calculated as $P_{appB>A}/P_{appA>B}$ for the condition with and without inhibitor. The net efflux ratio was determined by dividing the ER without inhibitor by the ER with inhibitor.

$$P_{app} = \frac{Vr}{S \times C_0} \times \frac{\Delta C_r}{\Delta t} \qquad (S1)$$

Where P_{app} is the apparent permeability in 10^{-6} cm/s, $\Delta C_r/\Delta t$ is the rate at which the compound appears in the receiver compartment (µM/s), C_0 is the amount of compound in the donor compartment at time point zero (µM), V_r is the volume of the receiver compartment (600 µL) and S is the surface area of the cell monolayer (0.33 cm^2).

The $CL_{efflux,vitro}$ was calculated using equation (2) [10]

$$CL_{efflux,vitro} = \frac{2\ x\ (ER - 1)\ x\ P_{app,A-B}\ x\ SA}{Pro_{cell}} \qquad (S2)$$

Where $CL_{efflux,vitro}$ is the *in vitro* efflux transporter-mediated clearance (µl/min/mg), ER is the efflux ratio, $P_{app,A\text{-}B}$ is the apparent passive permeability (cm/s) determined from inhibited MDCKII cells expressing BCRP or P-gp, SA is the surface area of the cell monolayer (0.33 cm^2) and Pro_{cell} is the protein amount of MDCKII-BCRP (34-75 µg) or P-gp (56-75 µg) cells in a 24-well transwell.

$CL_{efflux,vitro}$ was scaled to the whole-brain in vivo efflux transporter-mediated clearance ($CL_{efflux,vivo}$; µl/min/mg) by multiplying a relative activity factor (RAF), equation (3) [10]

$$\begin{aligned} CL_{efflux,vivo} &= CL_{efflux,vitro}\ x\ RAF \\ &= CL_{efflux,vitro}\ x\ \frac{Abundance\ in\ vivo}{Abundance\ in\ vitro}\ x\ BMvPGB\ x\ BW \qquad (S3) \end{aligned}$$

Where abundance *in vivo* represents the expression level in pmol/mg microvessels of 5.5 and 4.21[11-13] for BCRP and P-gp, respectively; *in vitro* represents the expression in pmol/mg cells of 13.18 for BCRP and 10.3 for P-gp (personal communication Jing Li, improved measurement of samples described in Li *et al.* 2017[10]); BMvPGB is the milligrams of brain microvessels per gram brain (0.244 mg protein/g brain), and BW is the brain weight (1400 gram)[10], resulting in a RAF of 143 for BCRP and a RAF of 140 for P-gp. Efflux transporter-mediated clearance at the BCSFB was not incorporated in the CNS model given the little information available on transporters abundance at this barrier.

Supplementary table S1| Summary of physicochemical, *in vitro*, and *in vivo* linezolid parameters

Parameter	Linezolid value
**Physicochemical properties**	
Molecular weight (g/mol)	337.35 [14]
$LogP_{o:w}$	0.232 [14]
Compound type	Monoprotic base [15]
pKa 1	1.7 [14, 15]
**Blood binding properties**	
Blood-to-plasma ratio	0.603 [14, 15]
Fraction unbound in plasma	0.69 [14, 15]
Plasma binding protein	Human serum albumin
**Absorption:** *First-Order Absorption Model* [a]	
f_a	1 [b]
k_a	0.9 [1]
fu_{Gut}	0.81 [c]
Q_{Gut} (L/h)	15.0 [c]
**Absorption:** *Advanced Dissolution, Absorption and Metabolism (ADAM) Model* [a]	
f_a	0.96 [c]
fu_{Gut}	0.81 [c]
Passive permeability (10^{-6} cm/s)	Linezolid: 9.7<br>Propranolol: 9.9<br>Scaler: 2.22
k_a	1.20
**Distribution:** *full PBPK Model*	
V_{ss} (L/kg) (Method 2: Rodgers *et al.*)	0.66 [16] for criticaly ill<br>Optimized for TB studies to 0.4
K_p scaler	1.7 for critically ill studies<br>1 for TB studies
**Elimination:** *enzyme kinetics*	
Additional clearance (L/h)	Reported plasma clearance per validation study [1, 3, 6, 17-22]
**Brain model**	
BBB	
PSB (L/h)	1.0, optimized [6]
$f_{u,br}$	0.81 [c]
$CL_{ABCG2, vitro}$ (µl/min/mg)	16; MDCK exp Eq 2
$CL_{ABCB1, vitro}$ (µl/min/mg)	2.1; MDCK exp Eq2
RAF for ABCG2	143
RAF for ABCB1	140
Blood-cranial CSF barrier	
PSC (L/h)	0.5 Assumed to be half of PSB [7]
fu_{CSF}	1 [23]
Brain-CSF barrier	
PSE (L/h)	300 [7]

[a] first order absorption model was applied to Alffenaar et al. (2010) and Garcia-Prats et al. (2019), the ADAM model was applied to Diacon et al. (2020); [b] default in Simcyp; [c] predicted by Simcyp

Supplementary Table S2| Overview of clinical studies used for the validation of the simulated PK curves.

Study	Dosing regimen (infusion time)	Patient no.	Age (years)	Indication (number of patients); Clearance applied (L/h)	Sample collection [a]	Males (%)	Reference	Corresponding figure
**Beer *et al.* 2007**	600 mg i.v. (0.5 h)	5	18-72	Subarachnoid hemorrhage (4)/ brain injury (1); Cl 13.7	Plasma, CSF	40	[17]	Supp. Figure 2
**Viaggi *et al.* 2011**	600 mg i.v. (1 h)	7	42-78	Subarachnoid (2) / intraventricular (2)/ intracerebral (5) hemorrhages; Cl 11.2	Plasma, CSF	71	[6]	
**Tsona *et al.* 2010** [b]	600 mg i.v. (0.5 h)	18	19-71	Hydrocephalus drainage (9)/ brain tumor excision (9); Cl 13.7 (Beer et al. 2007)	Plasma, CSF, brain	56	[24]	
**Yogev *et al.* 2010**	10 mg/kg i.v. (0.5 h)	10	0.25-21	Hydrocephalus drainage; Cl 13	Plasma, CSF	39	[20]	
**Beer *et al.* 2007**	600 mg i.v. BID (0.5 h)	5	18-72	Subarachnoid hemorrhage (4)/ brain injury (1); Cl 8	Plasma, CSF	40	[17]	Figure 1
**Luque *et al.* 2014**	600 mg i.v. BID (1 h)	11 (7)	39-74	Neurosurgical patients with (suspected) CNS infection; Cl 16.6	Plasma, CSF (ventricular (5) or lumbar (2) drain) [c]	64	[19]	
**Myrianthefs *et al.* 2006**	600 mg i.v. BID (1 h)	14 (9)	59±17	Neurosurgical patients with CNS infection (2) or prophylactic (12); Cl 7.3	Plasma, CSF (ventricular drain (9))	64	[18]	
**Viaggi *et al.* 2011**	600 mg i.v. BID (1 h)	7	42-78	Subarachnoid (2) / intraventricular (2)/ intracerebral (5) hemorrhages; Cl 13.4	Plasma, CSF	71	[6]	
**Yogev *et al.* 2010**	10 mg/kg i.v. BID (0.5 h)	10	0.25-21	Hydrocephalus drainage; Cl 11	Plasma, CSF	39	[20]	
**Alffenaar *et al.* 2010**	600 mg BID oral	14/12	24-36	MDR-TB [d]; Cl 3.95	Plasma	?	[1]	Figure 2
**Alffenaar *et al.* 2010**	600 mg BID oral	8	26-38	MDR-TB/ XDR-TB [d]; Cl 3.95	Plasma	50	[3]	
**Garcia-Prats *et al.* 2019**	~10 mg/kg BID oral	13	0.6-9.4	MDR-TB [d]; Cl 4.3	Plasma	76	[21]	
**Diacon *et al*. 2020**	300 mg BID oral	15	31±11	Drug-sensitive TB [e]; Cl 5.46	Plasma [f]	93	[22]	Figure 3
**Diacon *et al*. 2020**	1200 mg QD oral	16	34±14	Drug-sensitive TB [e]; Cl 4.9	Plasma [f]	87	[22]	

[a] CSF sample collection for all patients via ventricular drain unless otherwise stated [b] brain sample concentration from µg/g to µg/ml by multiplying by 1.1 gram/cm^3; [c] data could not be split between lumbar and cranial and were all included as cranial CSF values; [d] linezolid in addition to standard care; [e] linezolid monotherapy; [f] only PK parameters (no PK curves) reported, clearance was calculated by dividing dose by $AUC_{0\text{-}\tau}$, bioavailability is considered 100%

In case the maximum age was above 65, 65 was used as maximum in the simulation. BID: twice daily dosing; QD: once daily dosing

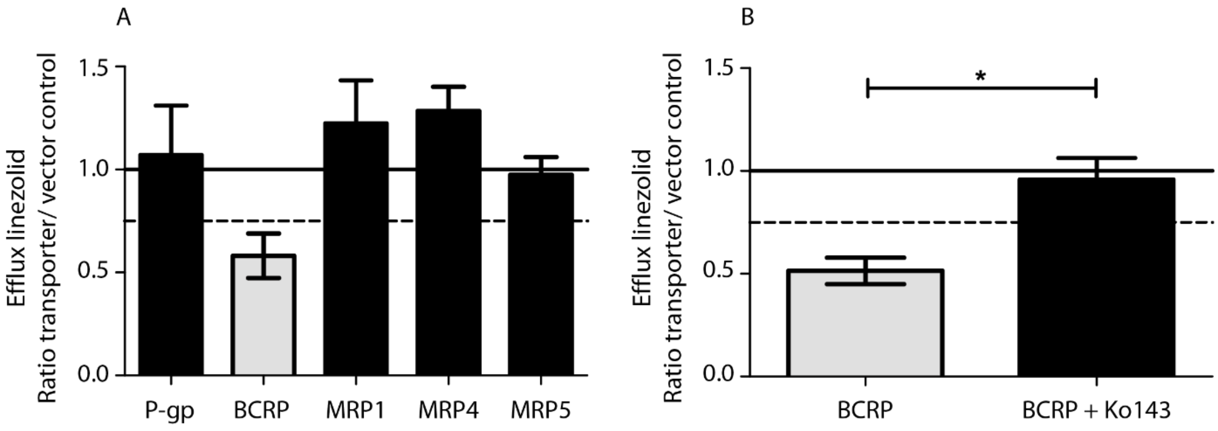

Supplementary Figure S1| Transport of linezolid in transiently transduced HEK293 cells by (A) efflux transporters present at the blood-brain barrier and for (B) BCRP with and without the BCRP inhibitor KO143 (1 µM). Dotted line represents a ratio between transporter and control of 0.75, the solid line a ratio of 1. Data are presented as mean with SEM of 3 independent experiments. *p<0.05 by a paired sampled T-test

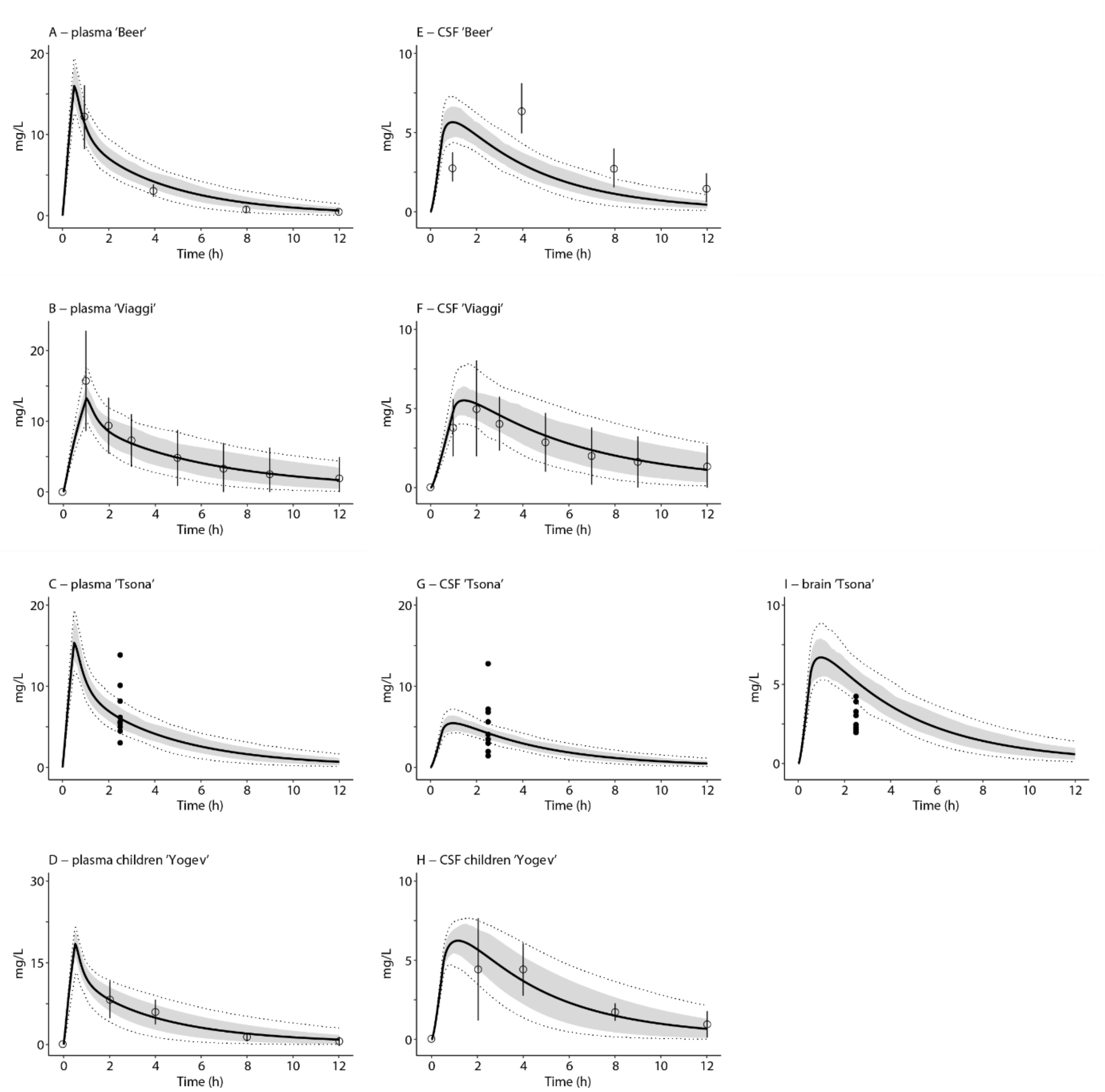

Supplementary Figure S2| Single dose simulations of 600 mg intravenous linezolid concentration-time profiles in plasma, cranial CSF and brain tissue in critically ill adult patients and plasma and cranial CSF after a single dose linezolid of 10 mg/kg in critically ill children. Solid black lines indicate simulation of the mean profile, the grey areas represent the area between the 16th and 84th percentile of the mean and the dotted lines indicate the 5th and 95th percentiles of the mean. Open circles indicate mean with standard deviation of the mean derived from literature [6, 17, 20].

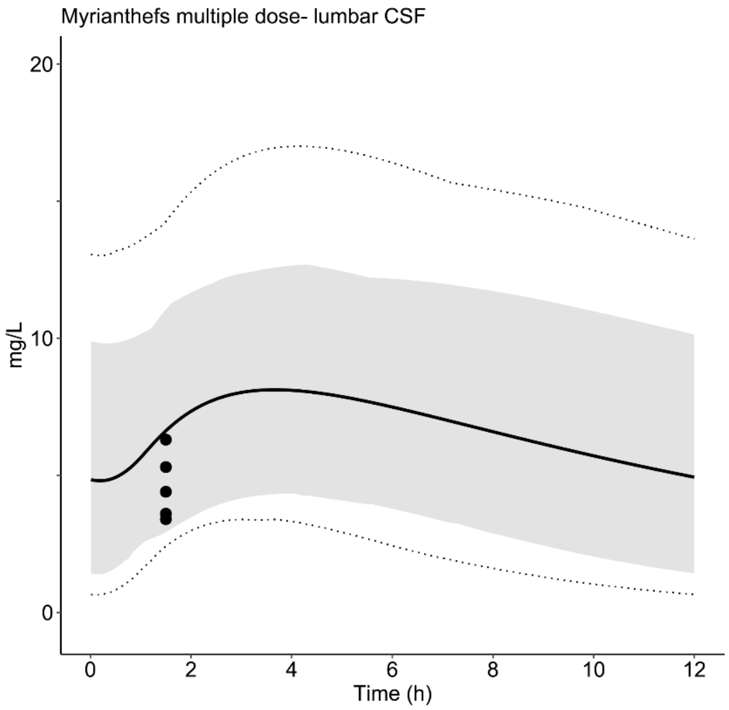

Supplementary Figure S3| Single dose simulations of 600 mg intravenous linezolid concentration-time profiles in spinal CSF in critically ill adult patients. Solid black line indicates simulation of the mean profile, the grey area represents the area between the 16th and 84th percentile of the mean and the dotted lines indicate the 5th and 95th percentiles of the mean. Closed dots indicate measured individual data derived from literature [18].

References

1. Alffenaar JW, Kosterink JG, van Altena R, van der Werf TS, Uges DR, Proost JH. Limited sampling strategies for therapeutic drug monitoring of linezolid in patients with multidrug-resistant tuberculosis. Ther Drug Monit 2010; 32: 97-101.
2. Rangaka MX, Cavalcante SC, Marais BJ, Thim S, Martinson NA, Swaminathan S, Chaisson RE. Controlling the seedbeds of tuberculosis: diagnosis and treatment of tuberculosis infection. Lancet 2015; 386: 2344-53.
3. Alffenaar JW, van Altena R, Harmelink IM, Filguera P, Molenaar E, Wessels AM, van Soolingen D, Kosterink JG, Uges DR, van der Werf TS. Comparison of the pharmacokinetics of two dosage regimens of linezolid in multidrug-resistant and extensively drug-resistant tuberculosis patients. Clin Pharmacokinet 2010; 49: 559-65.
4. Wynalda MA, Hauer MJ, Wienkers LC. Oxidation of the novel oxazolidinone antibiotic linezolid in human liver microsomes. Drug Metab Dispos 2000; 28: 1014-7.
5. Stalker DJ, Jungbluth GL, Hopkins NK, Batts DH. Pharmacokinetics and tolerance of single- and multiple-dose oral or intravenous linezolid, an oxazolidinone antibiotic, in healthy volunteers. J Antimicrob Chemother 2003; 51: 1239-46.
6. Viaggi B, Paolo AD, Danesi R, Polillo M, Ciofi L, Del Tacca M, Malacarne P. Linezolid in the central nervous system: comparison between cerebrospinal fluid and plasma pharmacokinetics. Scand J Infect Dis 2011; 43: 721-7.
7. Gaohua L, Neuhoff S, Johnson TN, Rostami-Hodjegan A, Jamei M. Development of a permeability-limited model of the human brain and cerebrospinal fluid (CSF) to integrate known physiological and biological knowledge: Estimating time varying CSF drug concentrations and their variability using in vitro data. Drug Metab Pharmacokinet 2016; 31: 224-33.
8. Gallagher E, Minn I, Chambers JE, Searson PC. In vitro characterization of pralidoxime transport and acetylcholinesterase reactivation across MDCK cells and stem cell-derived human brain microvascular endothelial cells (BC1-hBMECs). Fluids Barriers CNS 2016; 13: 10.
9. Mahnke H, Ballent M, Baumann S, Imperiale F, von Bergen M, Lanusse C, Lifschitz AL, Honscha W, Halwachs S. The ABCG2 Efflux Transporter in the Mammary Gland Mediates Veterinary Drug Secretion across the Blood-Milk Barrier into Milk of Dairy Cows. Drug metabolism and disposition: the biological fate of chemicals 2016; 44: 700-8.
10. Li J, Wu J, Bao X, Honea N, Xie Y, Kim S, Sparreboom A, Sanai N. Quantitative and Mechanistic Understanding of AZD1775 Penetration across Human Blood-Brain Barrier in Glioblastoma Patients Using an IVIVE-PBPK Modeling Approach. Clin Cancer Res 2017; 23: 7454-66.
11. Uchida Y, Ohtsuki S, Katsukura Y, Ikeda C, Suzuki T, Kamiie J, Terasaki T. Quantitative targeted absolute proteomics of human blood-brain barrier transporters and receptors. J Neurochem 2011; 117: 333-45.
12. Shawahna R, Uchida Y, Declèves X, Ohtsuki S, Yousif S, Dauchy S, Jacob A, Chassoux F, Daumas-Duport C, Couraud PO, Terasaki T, Scherrmann JM. Transcriptomic and quantitative proteomic analysis of transporters and drug metabolizing enzymes in freshly isolated human brain microvessels. Mol Pharm 2011; 8: 1332-41.
13. Al-Majdoub ZM, Al Feteisi H, Achour B, Warwood S, Neuhoff S, Rostami-Hodjegan A, Barber J. Proteomic Quantification of Human Blood-Brain Barrier SLC and ABC Transporters in Healthy Individuals and Dementia Patients. Mol Pharm 2019; 16: 1220-33.
14. Duan P, Fisher JW, Yoshida K, Zhang L, Burckart GJ, Wang J. Physiologically Based Pharmacokinetic Prediction of Linezolid and Emtricitabine in Neonates and Infants. Clin Pharmacokinet 2017; 56: 383-94.
15. Gandelman K, Zhu T, Fahmi OA, Glue P, Lian K, Obach RS, Damle B. Unexpected effect of rifampin on the pharmacokinetics of linezolid: in silico and in vitro approaches to explain its mechanism. J Clin Pharmacol 2011; 51: 229-36.
16. Pfizer Inc. (per FDA). Product Information: ZYVOX(R) intravenous injection, oral tablets suspension, linezolid intravenous injection, oral tablets suspension. New York, NY, 2013.

17. Beer R, Engelhardt KW, Pfausler B, Broessner G, Helbok R, Lackner P, Brenneis C, Kaehler ST, Georgopoulos A, Schmutzhard E. Pharmacokinetics of intravenous linezolid in cerebrospinal fluid and plasma in neurointensive care patients with staphylococcal ventriculitis associated with external ventricular drains. Antimicrob Agents Chemother 2007; 51: 379-82.
18. Myrianthefs P, Markantonis SL, Vlachos K, Anagnostaki M, Boutzouka E, Panidis D, Baltopoulos G. Serum and cerebrospinal fluid concentrations of linezolid in neurosurgical patients. Antimicrob Agents Chemother 2006; 50: 3971-6.
19. Luque S, Grau S, Alvarez-Lerma F, Ferrandez O, Campillo N, Horcajada JP, Basas M, Lipman J, Roberts JA. Plasma and cerebrospinal fluid concentrations of linezolid in neurosurgical critically ill patients with proven or suspected central nervous system infections. Int J Antimicrob Agents 2014; 44: 409-15.
20. Yogev R, Damle B, Levy G, Nachman S. Pharmacokinetics and distribution of linezolid in cerebrospinal fluid in children and adolescents. Pediatr Infect Dis J 2010; 29: 827-30.
21. Garcia-Prats AJ, Schaaf HS, Draper HR, Garcia-Cremades M, Winckler J, Wiesner L, Hesseling AC, Savic RM. Pharmacokinetics, optimal dosing, and safety of linezolid in children with multidrug-resistant tuberculosis: Combined data from two prospective observational studies. PLoS Med 2019; 16: e1002789.
22. Diacon AH, De Jager VR, Dawson R, Narunsky K, Vanker N, Burger DA, Everitt D, Pappas F, Nedelman J, Mendel CM. Fourteen-Day Bactericidal Activity, Safety, and Pharmacokinetics of Linezolid in Adults with Drug-Sensitive Pulmonary Tuberculosis. Antimicrob Agents Chemother 2020; 64.
23. Tsuji Y, Hiraki Y, Matsumoto K, Mizoguchi A, Sadoh S, Kobayashi T, Takemura Y, Sakamoto S, Morita K, Kamimura H, Karube Y. Pharmacokinetics and protein binding of linezolid in cerebrospinal fluid and serum in a case of post-neurosurgical bacterial meningitis. Scand J Infect Dis 2011; 43: 982-5.
24. Tsona A, Metallidis S, Foroglou N, Selviaridis P, Chrysanthidis T, Lazaraki G, Papaioannou M, Nikolaidis J, Nikolaidis P. Linezolid penetration into cerebrospinal fluid and brain tissue. J Chemother 2010; 22: 17-9.
